# Fulminant Mantle Cell Lymphoma Presenting With Hepatic Infiltration and Spontaneous Tumor Lysis Syndrome

**DOI:** 10.7759/cureus.110249

**Published:** 2026-06-04

**Authors:** Shyamal Sheth, Amer Alsamman

**Affiliations:** 1 Internal Medicine, University of Chicago, Chicago, USA; 2 Gastroenterology and Hepatology, Franciscan Health Olympia Fields, Olympia Fields, USA

**Keywords:** hepatic involvement, infiltrative lymphoma, mantle cell lymphoma, non-hodgkin lymphoma, tumor lysis syndrome

## Abstract

Mantle cell lymphoma (MCL) is an uncommon, aggressive B-cell non-Hodgkin lymphoma that typically presents with lymphadenopathy, splenomegaly, and gastrointestinal (GI) tract involvement, most often as multiple lymphomatous polyps. While hepatic involvement by MCL is rare and usually asymptomatic, hepatic lesions in clinical practice more often represent metastases from GI, breast, lung, or pancreatic primaries.

We report a case of MCL initially presenting with tumor lysis syndrome (TLS) and cholestatic liver injury, mimicking hepatobiliary malignancy. A 90-year-old male with coronary artery disease complicated by coronary artery bypass grafting presented with abdominal pain, nausea, and vomiting. He was initially discharged with a presumed diagnosis of chronic lymphocytic leukemia based on the complete blood count. He re-presented several days later with worsening jaundice, anorexia, weakness, and a shuffling gait. Laboratory evaluation revealed leukocytosis, an acute kidney injury, hyperkalemia, hyperuricemia, hyperammonemia, and a cholestatic pattern of liver injury. Cross-sectional imaging demonstrated hepatosplenomegaly, multifocal splenic infarcts, gallbladder wall thickening with cholelithiasis, and no biliary ductal dilation. Bone marrow flow cytometry revealed a CD5+ (bright), CD23+, FMC7+, CD10−, kappa-restricted mature B-cell population consistent with MCL. Liver biopsy confirmed diffuse hepatic infiltration by MCL with a high proliferative index (Ki-67: 70-80%). Given the presentation of spontaneous TLS, the patient was admitted to the intensive care unit for vasopressors, continuous renal replacement therapy, and rasburicase. Given poor functional status, curative therapy was deferred, and palliative care was initiated. The patient ultimately passed away from multi-organ failure.

This case underscores the importance of considering infiltrative lymphoma in patients presenting with hepatosplenomegaly, atypical cholestasis, and no clear obstructive process. Early liver biopsy can be pivotal in differentiating hematologic malignancy from primary hepatobiliary carcinoma when imaging is inconclusive. Although hepatic MCL is rare, it can present with fulminant disease and spontaneous TLS. Timely recognition may expedite diagnosis and guide appropriate management, especially in elderly or frail patients where treatment must be individualized.

## Introduction

Mantle cell lymphoma (MCL) is an aggressive mature B-cell non-Hodgkin lymphoma (NHL) accounting for approximately 6% of NHL diagnoses in the United States [1]. It predominantly affects older men, with a median age at diagnosis of 60-65 years and a male-to-female ratio of approximately 3:1 [2]. MCL is defined by the t(11;14)(q13;q32) translocation, which drives overexpression of cyclin D1 and dysregulated cell-cycle progression [3]. Most patients present with peripheral lymphadenopathy, splenomegaly, and bone marrow involvement; gastrointestinal involvement, typically as multiple lymphomatous polyps, is seen in up to 30% of cases [4].

For gastroenterologists and hepatologists, an underappreciated diagnostic challenge is that cholestatic liver injury in the absence of biliary obstruction can be the presenting manifestation of an infiltrative hematologic malignancy. Although hepatic involvement by MCL has been reported in up to 50% of autopsy series, it is rarely symptomatic and seldom the presenting feature [5]. When it does occur, MCL tends to infiltrate the liver diffusely rather than form discrete masses, mimicking primary hepatobiliary pathology on imaging and obscuring the underlying diagnosis [6]. Spontaneous tumor lysis syndrome (TLS), arising from intrinsic tumor proliferation rather than cytotoxic therapy, is an exceptionally rare complication of MCL that portends an extremely poor prognosis [7].

We report a 90-year-old male who presented with cholestatic jaundice, hepatosplenomegaly, and spontaneous TLS, initially raising concern for hepatobiliary malignancy, but ultimately diagnosed with MCL via bone marrow flow cytometry and liver biopsy. This case highlights two clinically actionable lessons relevant to gastroenterologists and hepatologists: cholestatic liver injury without biliary obstruction should prompt consideration of infiltrative lymphoma, and early liver biopsy can be definitive when imaging is non-diagnostic.

This article was presented at ACG 2025, where it won the Presidential Award for novel clinical findings.

## Case presentation

A 90-year-old male, with a significant past medical history for coronary artery disease complicated by coronary artery bypass grafting and known pancreatic cysts, presented to the emergency department with a several-day history of abdominal pain, nausea, and vomiting. During this initial presentation, laboratory evaluation revealed leukocytosis, and peripheral blood smear findings were suggestive of chronic lymphocytic leukemia (CLL). Imaging on admission revealed a splenic infarct. Labs on admission were only significant for an acute kidney injury secondary to volume loss. The patient was discharged after significant clinical improvement with supportive medical management. Outpatient hematology follow-up was arranged. 

He re-presented to our institution several days later with progressive jaundice, acute weight loss, generalized weakness, and a new-onset shuffling gait. On arrival, he was found to be hemodynamically unstable with hypotension (87/54) and mild hypoxemia (82% SpO2) requiring supplemental oxygen. Laboratory evaluation on re-presentation is shown in Table 1. 

**Table 1:** Relevant laboratory values on admission AST: Aspartate aminotransferase; ALT: alanine transaminase

Laboratory Parameter (Reference Range)	Patient Value	Clinical Significance
Hematologic		
White blood cell count (5.0–11.0 × 10⁹/L)	109.5 × 10⁹/L	Marked leukocytosis with lymphocyte predominance
Renal/Metabolic		
Creatinine (0.8–1.3 mg/dL)	3.1 mg/dL	Acute kidney injury (baseline unknown)
Potassium (3.5–5.0 mmol/L)	9.1 mmol/L	Life-threatening hyperkalemia
Tumor lysis markers		
Uric acid (4.0–8.5 mg/dL)	16.2 mg/dL	Marked hyperuricemia
Ammonia (10–37 µmol/L)	82 µmol/L	Hyperammonemia
Hepatobiliary		
Total bilirubin (0.1–1.2 mg/dL)	19.7 mg/dL	Severe hyperbilirubinemia
Direct bilirubin (0.0–0.3 mg/dL)	12.6 mg/dL	Predominantly conjugated
Alkaline phosphatase (44–147 U/L)	472 U/L	Cholestatic pattern
AST (8–48 U/L)	59 U/L	Mild hepatocellular injury
ALT (7–55 U/L)	101 U/L	Mild hepatocellular injury
Albumin (3.5–5.0 g/dL)	3.6 g/dL	Within the normal range

**Figure 1:**
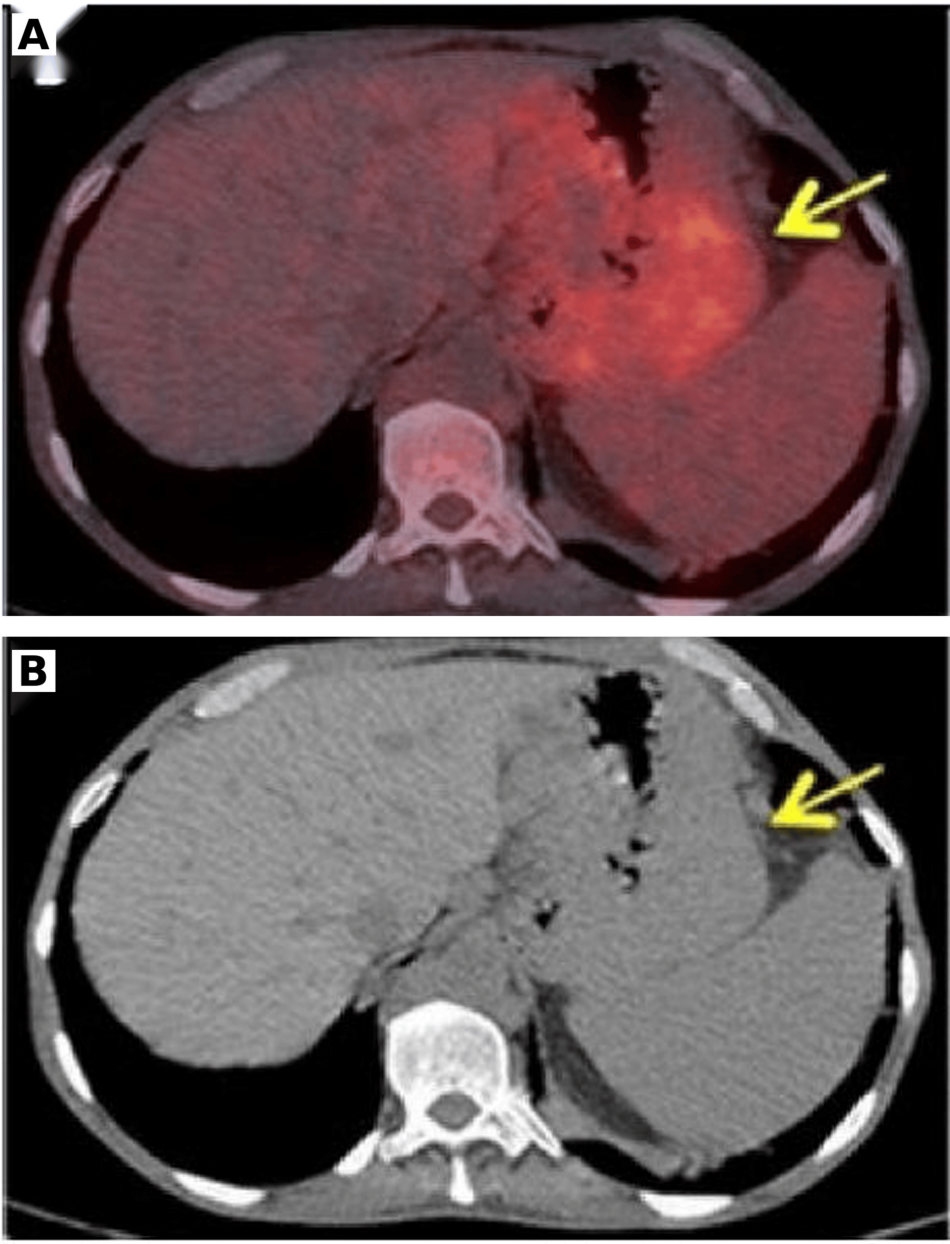
Cross-sectional imaging of the abdomen demonstrating hepatosplenomegaly. (A) Axial PET/CT fusion image showing focal FDG-avid uptake in the spleen (yellow arrow), with diffusely enlarged liver and spleen. (B) Corresponding axial non-contrast CT image at the same level demonstrating splenomegaly with a hypodense splenic lesion consistent with infarction (yellow arrow). No biliary ductal dilation was identified

Based on the presenting labs and clinical picture, the patient was found to be in Grade 3 Tumor Lysis based on the Cairo-Bishop scale [8]. Cross-sectional imaging, including computed tomography (CT) of the abdomen and pelvis and magnetic resonance imaging (MRI)/magnetic resonance cholangiopancreatography (MRCP), demonstrated hepatosplenomegaly with multifocal splenic infarcts, gallbladder wall thickening with cholelithiasis, and, notably, no evidence of intrahepatic or extrahepatic biliary ductal dilation (Figures 1A, 1B). The absence of biliary obstruction on imaging was pivotal in redirecting the diagnostic workup away from obstructive biliary pathology. He required transfer to the intensive care unit (ICU) for hemodynamic monitoring and was managed with vasopressor support, continuous renal replacement therapy (CRRT), and rasburicase for uric acid reduction.

Given the constellation of massive hepatosplenomegaly, cholestatic liver injury without biliary obstruction, and extreme leukocytosis, an infiltrative hematologic malignancy was strongly suspected. Bone marrow biopsy with flow cytometry was performed and revealed a CD5+ (bright), CD23+, FMC7+, CD10−, kappa light chain-restricted mature B-cell population. This immunophenotype, particularly the bright CD5 expression, FMC7 positivity, and CD10 negativity, was consistent with MCL rather than CLL (Table 2).

**Table 2:** Bone marrow flow cytometry immunophenotype MCL: Mantle cell lymphoma; CLL: chronic lymphocytic leukemia

Marker	Result	Interpretation
CD19	Positive	Pan B-cell marker; confirms B-cell lineage
CD20	Positive	Pan B-cell marker; confirms B-cell lineage
CD5	Positive (bright)	Aberrant expression; seen in MCL and CLL
CD23	Positive	Variable in MCL; classically positive in CLL
FMC7	Positive	Favors MCL; typically negative in CLL
CD10	Negative	Excludes follicular lymphoma
Kappa light chain	Restricted	Monoclonal B-cell population

**Figure 2:**
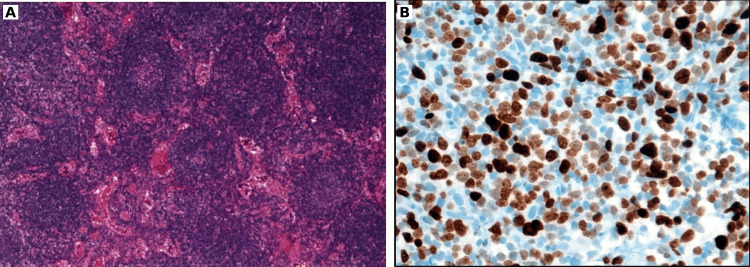
Histopathology of the liver biopsy. (A) Hematoxylin and eosin stain showing diffuse infiltration of the hepatic parenchyma by a monomorphic population of small-to-medium-sized lymphoid cells, consistent with mantle cell lymphoma. (B) Ki-67 immunohistochemistry demonstrating a high proliferative index of approximately 70–80%, indicative of aggressive disease biology

Liver biopsy was subsequently performed and confirmed diffuse hepatic parenchymal infiltration by MCL. Immunohistochemistry demonstrated a high proliferative index with Ki-67 staining of 70-80%, indicative of aggressive disease biology (Figures 2A, 2B). 

Given the patient’s advanced age (90 years), multiple comorbidities, severely compromised functional status, and the aggressive nature of his disease (high Ki-67, spontaneous TLS, multi-organ dysfunction), a goals-of-care discussion was held with the patient and his family. The hematology-oncology team determined that the risks of cytotoxic chemotherapy outweighed potential benefits. The decision was made to transition to comfort-focused palliative care. The patient subsequently died from progressive multi-organ failure during hospitalization.

## Discussion

This case illustrates a diagnostically challenging presentation of MCL with predominant hepatic involvement masquerading as hepatobiliary malignancy. Several features of this case merit focused discussion.

MCL with primary hepatic presentation is exceptionally uncommon. While post-mortem studies suggest hepatic involvement in up to 50% of MCL cases, clinically apparent hepatic disease as the presenting manifestation is rare [5,6]. When it does occur, the pattern of injury, diffuse infiltration without discrete mass formation, closely simulates primary hepatocellular carcinoma, cholangiocarcinoma, or metastatic disease, particularly in elderly patients with other risk factors for hepatobiliary pathology such as cholelithiasis or pancreatic cysts, as seen in our patient.

The cholestatic pattern of liver injury in the absence of biliary obstruction is a key finding that should raise clinical suspicion for infiltrative disease. Infiltrative processes, including lymphoma, sarcoidosis, amyloidosis, and granulomatous hepatitis, can disrupt intrahepatic bile canalicular transport mechanisms, leading to disproportionate elevations in ALP and bilirubin with minimal transaminase elevation [9]. In our patient, the total bilirubin of 19.7 mg/dL with an ALP of 472 U/L and only mild AST elevation of 59 U/L, combined with a normal biliary tree on MRI/MRCP, strongly supported an infiltrative etiology.

Early liver biopsy proved critical in this case. When imaging is inconclusive and infiltrative disease is suspected, tissue diagnosis via liver biopsy can provide rapid and definitive pathological characterization. Prior case reports have similarly highlighted that liver biopsy, rather than repeat cross-sectional imaging or empirical treatment, is often the most efficient route to diagnosis in patients with hepatosplenomegaly and atypical cholestasis [9,10]. The high Ki-67 proliferative index (70-80%) identified on our patient’s liver biopsy confirmed the aggressive nature of the disease and aligned with the fulminant clinical trajectory.

This case also illustrates the diagnostic limitations of relying on peripheral smear morphology alone to distinguish CLL from other CD5-positive B-cell lymphoproliferative disorders. At our patient’s initial presentation, leukocytosis with lymphocyte predominance and a peripheral smear showing small mature-appearing lymphocytes supported a presumptive diagnosis of CLL. This is a reasonable initial impression given that CLL accounts for the majority of CD5+ B-cell lymphoproliferative disorders in older adults. Given his advanced age and comorbidities, definitive flow cytometric subtyping was not pursued at that time, and the patient was discharged for outpatient hematology follow-up. This decision proved consequential when he re-presented days later with fulminant disease. MCL and CLL share several morphologic and immunophenotypic features: small-to-medium lymphocytes on smear and CD5 positivity by flow, but their clinical trajectories diverge dramatically. The distinguishing markers require flow cytometric evaluation: MCL typically demonstrates bright CD5, FMC7 positivity, and CD23 negativity or variable expression, whereas CLL classically shows dim CD5, FMC7 negativity, and bright CD23. Of note, our patient’s CD23-positive immunophenotype reflects an increasingly recognized CD23+ MCL variant that can mimic CLL even more closely on flow cytometry, further complicating distinction; in this context, cyclin D1 by immunohistochemistry or t(11;14) detection by fluorescence in situ hybridization remains confirmatory and should be pursued when the clinical course is discordant with an indolent diagnosis. For elderly patients with unexplained lymphocytosis and a CD5+ B-cell phenotype suspected on smear, early flow cytometry with cyclin D1 or FISH confirmation is warranted even when aggressive therapy is unlikely to be offered, because the correct diagnosis fundamentally shapes prognosis, surveillance, and goals-of-care discussions. 

The development of spontaneous TLS in MCL is an uncommon but life-threatening complication. TLS most commonly occurs as a consequence of cytotoxic therapy; however, spontaneous TLS arising from rapid intrinsic tumor cell turnover has been described in hematologic malignancies with high proliferative burden [7,11,12]. A Ki-67 index exceeding 70%, as seen in our case, reflects a near-exponential rate of tumor proliferation and can exceed the body’s metabolic capacity to clear the byproducts of cellular breakdown. The resulting hyperuricemia, hyperkalemia, and oliguric AKI in our patient required aggressive ICU-level management including CRRT and rasburicase.

From an oncologic standpoint, MCL in a 90-year-old patient with multiple comorbidities and spontaneous TLS presenting with multi-organ dysfunction poses an extraordinary treatment challenge. Standard MCL therapy including chemoimmunotherapy (e.g., R-CHOP, R-HyperCVAD) or targeted agents such as ibrutinib carries significant toxicity in frail elderly patients [12]. The Comprehensive Geriatric Assessment (CGA) or simplified frailty scoring tools, such as the Clinical Frailty Scale, can assist in estimating treatment tolerance and expected outcomes, but in cases of pre-treatment multi-organ failure, curative intent is often not feasible [13,14]. Our case underscores the importance of early goals-of-care discussions in patients with high-grade lymphoma and severe functional compromise.

For gastroenterologists specifically, this case reinforces several teaching points: hepatosplenomegaly with a cholestatic biochemical profile in the absence of biliary obstruction should prompt consideration of infiltrative hematologic malignancy; early liver biopsy is a high-yield, low-delay pathway to diagnosis in this clinical scenario; and an initial peripheral blood smear or flow cytometry finding of a lymphoproliferative disorder even if labeled as a less aggressive entity such as CLL should be revisited if the clinical course escalates unexpectedly.

## Conclusions

Primary hepatic involvement in MCL is rare but can manifest as an aggressive, fulminant disease mimicking hepatobiliary malignancy. Our case demonstrates that atypical cholestasis with hepatosplenomegaly and no biliary obstruction should prompt consideration of infiltrative lymphoma on the differential diagnosis. Early liver biopsy is a critical and efficient diagnostic tool in this setting. The presence of spontaneous TLS, a consequence of high tumor proliferative burden, markedly worsens prognosis and demands urgent ICU-level intervention. In elderly or frail patients, individualized, goal-aligned management is paramount. Heightened awareness of this rare but serious presentation may facilitate earlier diagnosis and more informed clinical decision-making.

## References

[1] Swerdlow SH, Campo E, Pileri SA (2016). The 2016 revision of the World Health Organization classification of lymphoid neoplasms. Blood.

[2] Vose JM (2017). Mantle cell lymphoma: 2017 update on diagnosis, risk-stratification, and clinical management. Am J Hematol.

[3] Jares P, Colomer D, Campo E (2012). Molecular pathogenesis of mantle cell lymphoma. J Clin Invest.

[4] Romaguera JE, Medeiros LJ, Hagemeister FB (2003). Frequency of gastrointestinal involvement and its clinical significance in mantle cell lymphoma. Cancer.

[5] Argatoff LH, Connors JM, Klasa RJ, Horsman DE, Gascoyne RD (1997). Mantle cell lymphoma: a clinicopathologic study of 80 cases. Blood.

[6] Rowbotham D, Wendon J, Williams R (1998). Acute liver failure secondary to hepatic infiltration: a single centre experience of 18 cases. Gut.

[7] Kekre N, Djordjevic B, Touchie C (2012). Spontaneous tumour lysis syndrome. CMAJ.

[8] Cairo MS, Bishop M (2004). Tumour lysis syndrome: new therapeutic strategies and classification. Br J Haematol.

[9] Hilscher MB, Kamath PS, Eaton JE (2020). Cholestatic liver diseases: a primer for generalists and subspecialists. Mayo Clin Proc.

[10] Williams MO, Akhondi H, Khan O (2019). Primary hepatic follicular lymphoma presenting as sub-acute liver failure: a case report and review of the literature. Clin Pathol.

[11] Howard SC, Jones DP, Pui CH (2011). The tumor lysis syndrome. N Engl J Med.

[12] Dreyling M, Klapper W, Rule S (2018). Blastoid and pleomorphic mantle cell lymphoma: still a diagnostic and therapeutic challenge!. Blood.

[13] Rubenstein LZ, Josephson KR, Wieland GD, English PA, Sayre JA, Kane RL (1984). Effectiveness of a geriatric evaluation unit. A randomized clinical trial. N Engl J Med.

[14] Rockwood K, Song X, MacKnight C, Bergman H, Hogan DB, McDowell I, Mitnitski A (2005). A global clinical measure of fitness and frailty in elderly people. CMAJ.

